# Enzyme-Digested Peptides Derived from *Lates calcarifer* Enhance Wound Healing after Surgical Incision in a Murine Model

**DOI:** 10.3390/md19030154

**Published:** 2021-03-16

**Authors:** Yen-An Lin, Pei-Yi Chu, Wen-Lung Ma, Wei-Chung Cheng, Shu-Ting Chan, Juan-Cheng Yang, Yang-Chang Wu

**Affiliations:** 1 Graduate Institute of Basic Medical Science, School of China Medical University, Taichung 40402, Taiwan; riinryxc@gmail.com; 2 Graduate Institute of Biomedical Sciences, China Medical University, Taichung 40402, Taiwan; maverick@mail.cmu.edu.tw (W.-L.M.); cwc0702@gmail.com (W.-C.C.); 3 Chinese Medicine Research and Development Center, China Medical University Hospital, Taichung 40402, Taiwan; ositachucmu@gmail.com; 4 Research Center for Tumor Medical Science, China Medical University, Taichung 40402, Taiwan; 5 TCI Academy, TCI CO., Ltd., Taipei 11448, Taiwan; Rebecca.Chan@tci-bio.com; 6 Graduate Institute of Integrated Medicine, China Medical University, Taichung 40402, Taiwan; 7 Department of Medical Laboratory Science and Biotechnology, College of Medical and Health Science, Asia University, Taichung 41354, Taiwan

**Keywords:** *Lates calcarifer*, wound healing, angiogenesis, murine model

## Abstract

Surgical wounds are common injuries of skin and tissues and usually become a clinical problem. Until now, various synthetic and natural peptides have been widely explored as potential drug candidates for wound healing. Inhibition of the TNF-α signaling pathway and promotion of angiogenesis are suggested to be involved in their effects. Angiogenesis at the wound site is one of the essential requisites for rapid healing. In the present study, a novel peptide extract derived from the natural source *Lates calcarifer*, commonly known as sea bass or barramundi, was evaluated for its wound healing property. The specific acidic and enzymatic approaches were employed for producing sea bass extract containing small size peptides (molecular weight ranging from 1 kD to 5 kD). The cytotoxicity of the extract was examined in HaCaT and NIH3T3. After this, the effects of enzyme digested peptide extracts of sea bass on wound healing in mice were investigated. The peptide extracts (660 and 1320 mg/kg/day) and control protein (1320 mg/kg/day) was orally given to the wounded mice, respectively, for 12 days. The surgical method was improved by implanting a silicone ring at the wound site. The ring avoided the contracting effect in murine wounds, making it more closely related to a clinical condition. The results showed promising improvement at the wound site in mice. Sea bass peptide extracts accelerated the wound healing process and enhanced the microvessel formation at the wound site. The remarkable effects of this novel sea bass peptide extract in healing traumatic injuries revealed a new option for developing wound management.

## 1. Introduction

Fisheries byproducts have been used to make highly profitable products for improving human health. It is widely considered that murine organisms serve as a rich source of a variety of essential components along with nutrients, such as collagen, gelatin, polyunsaturated fatty acids (PUFA), vitamins, antioxidants, enzymes, and bioactive peptides that can aid the wound healing process [1]. Many studies revealed numerous health benefits, food applications, and bioactivities of fish byproducts. For instance, flounder byproducts enhance antioxidant activity, and some collagen and gelatin-related peptides extracted from tilapia assist in wound-healing treatments [1,2]. With ongoing technological advancements, identification and isolation of the essential components in fish byproducts are accelerated. Numerous fisheries byproducts are being explored for their intrinsic, essential property towards wound healing in recent years [3]. Further, the components of fish byproducts, such as the ratio of various peptides, collagens and gelatins, are especially abundant and unique [4].

Sea bass, a nutritious and valuable fish, is mainly distributed in the shallow waters of Japan, North Korea, and China, which is one of the most important commercial marine species cultured in Taiwan [5,6]. Chinese traditional medicine books also recorded that sea bass is beneficial for muscle condition, blood and spleen enrichment, and general health [7]. Sea bass has high nutritional value, being rich in digestible protein, fat, vitamin A, vitamin B2, inorganic salts, sugars, niacin, calcium, phosphorus, potassium, zinc, copper, iron, selenium, and other nutrients [8]. Taiwanese like to have sea bass soup after surgery or after giving birth. Because sea bass meat and skin are rich in protein and colloids and can be supplemented with other nutrients. The elements in sea bass also possess the angiogenesis promoting property [9,10]. It is believed that sea bass can improve circulation, heal wounds, and restore tissue. Several studies have revealed that bioactive peptides and some small peptides promote wound healing and microvascular morphogenesis [11,12,13,14,15]. However, the effect of sea bass-derived small peptides on wound healing is still unclear.

The wound healing process mainly consists of three stages: inflammation, proliferation, and remodeling [16,17]. During the coagulation and inflammatory phase, macrophages release transforming growth factor-β and its related reactive oxygen species, which in turn stimulate the marginalization and migration of white blood cells and vascular endothelial cells. Then, in the hyperplastic remodeling phase, fibroblasts, keratinocytes, and endothelial cells play an important role in wound healing and affect subsequent collagen renewal and remodeling normalization. Incision models using rodents have been commonly conducted for wound healing research over the past half-century due to their availability, low-cost, and ease of use [18,19,20]. However, the main wound healing mechanism in rodents is skin contraction. In humans, re-epithelialization and granulation tissue formation are the main mechanisms. Therefore, the structural and healing differences between human and animal skin constitute the major problem in wound healing study, which may lead to evaluation error on the wound healing treating agents. To solve this problem, we established a splint wound model to avoid contraction that has greater similarity to the human condition [21].

The aim of the study is to investigate the effects of sea bass-derived small peptides on wound healing in mice. In this study, we derived the *Lates calcarifer* peptide extraction by enzymatic hydrolysis method. The effects of sea bass peptides on keratinocytes HaCaT and fibroblasts NIH3T3 viability and on wound healing in splint wound model were investigated.

## 2. Results

### 2.1. Lates Calcarifer-Extracted Peptide (LCEP) Molecular Weight Analysis and Comparison

**Figure 1:**
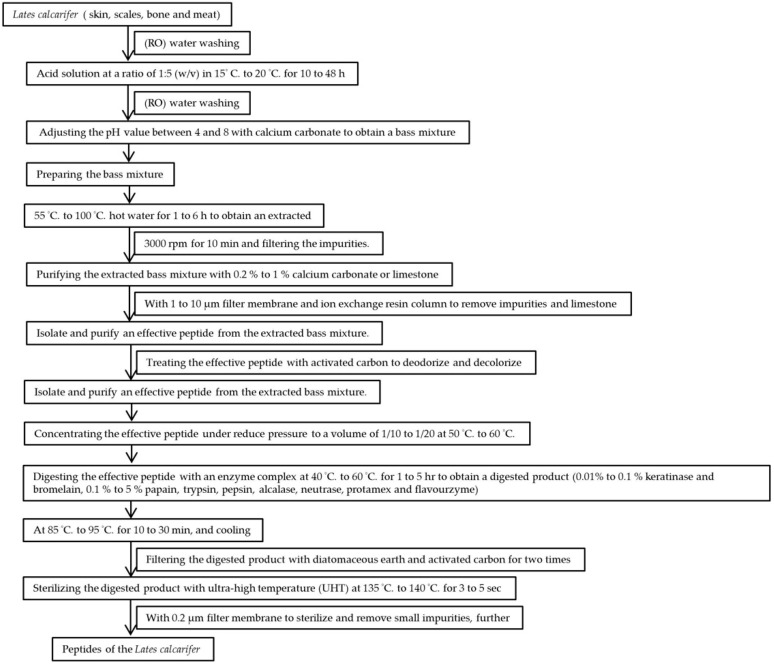
The procedure of *Lates calcarifer*-extracted peptide (LCEP) production.

**Figure 2:**
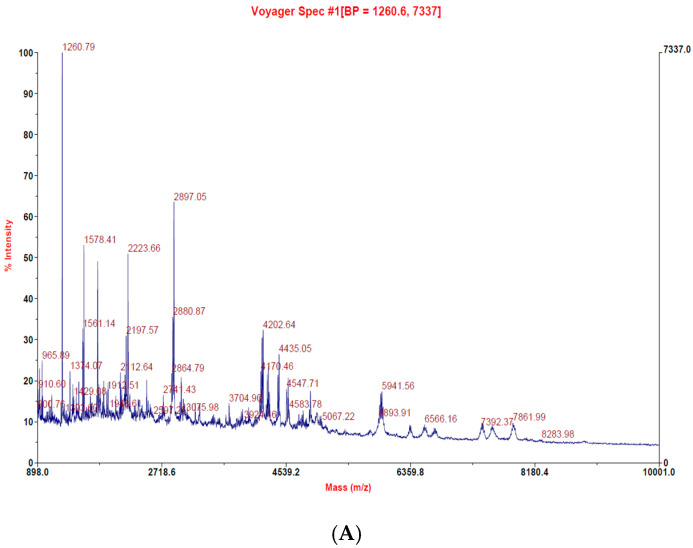

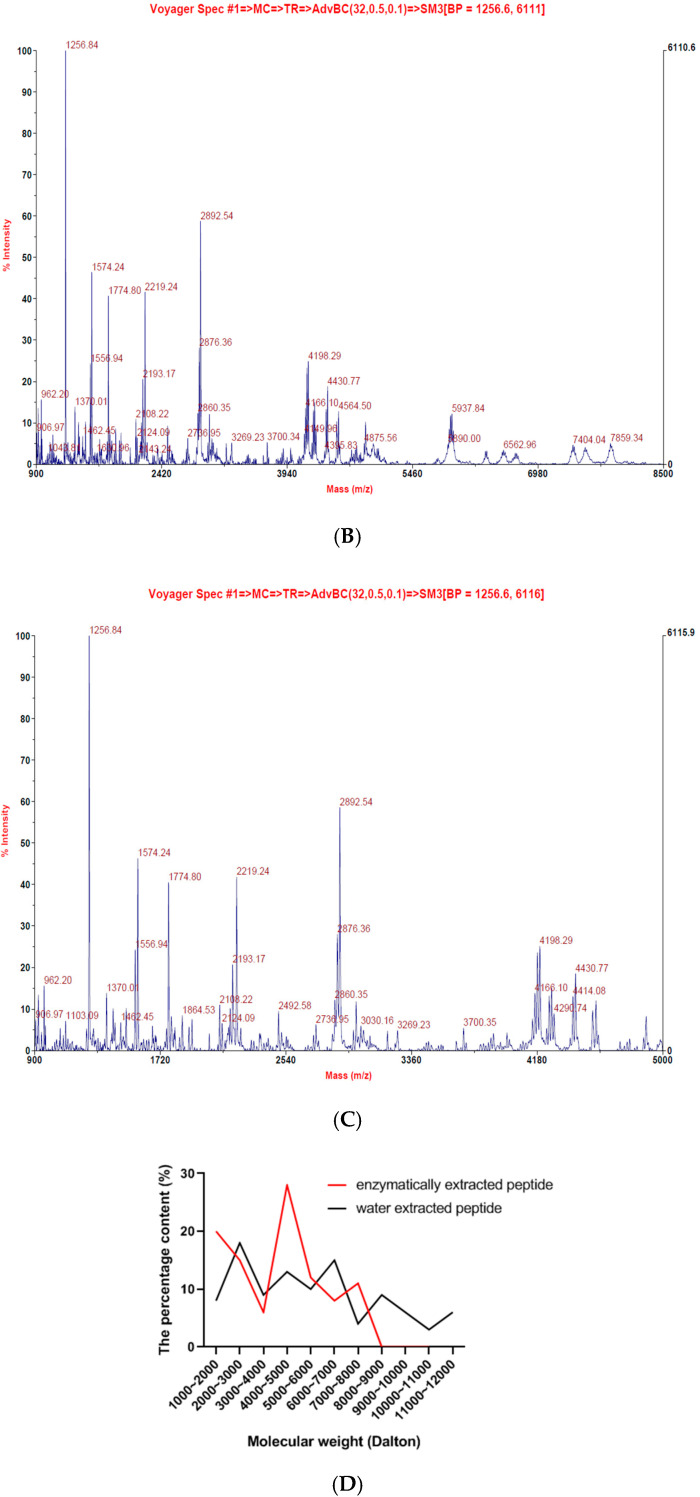
Molecular weight distribution of peptide extract derived from sea bass. LC–MS/MS was used to determine the molecular weight and analysis the molecular weight distribution of sea bass peptide extract. (**A**) 10,000 mass (*m*/*z*), (**B**) 8500 mass (*m*/*z*), (**C**) 5000 mass (*m*/*z*), (**D**) molecular weight distribution of enzymatically extracted peptide (red line) and nonenzymatically extracted peptide (black line).

We have followed the patented fermentation process (US patent number: US10,005,823 B2) for the extraction of sea bass peptides (Figure 1). The patented procedure increased the yield of small peptides in comparison to macropeptides. There were 20 small peptides obtained, which were further concentrated, and the molecular weight was found below 5000 mass (*m*/*z*) (Figure 2A–C). The increase in small peptide production from sea bass by utilizing the patented process was around 21%, while reduces the macropeptides by 22%. Compared with sea bass soup with macromolecular peptides, LCEP obtained by the patented method was concentrated with more small peptides (Figure 2D). It may be more beneficial for absorption as well as wound healing.

### 2.2. In Vitro Toxicity Analysis of LCEP

**Figure 3:**
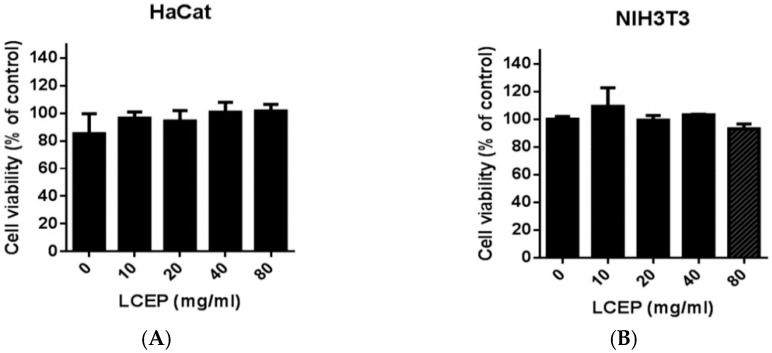
Effects of sea bass peptide extract on cell viability. HaCaT (**A**) and NIH3T3 (**B**) cells were treated with *Lates calcarifer* extract peptide (LCEP) 0, 10, 20, 40 and 80 mg/mL, respectively. Viabilities were determined at 24 h. Data are means ± standard deviation (SD) (*n* = 9). There is no statistically significant difference between the groups.

Although extracts from fish are generally safe and acceptable, the toxicity is not well-defined. Therefore, we examined the cytotoxicity of LCEP in the HaCaT cell line, which is an immortal keratinocyte cell derived from human skin that is widely applied in skin research. The MTT assay was conducted for the toxicity assessment of LCEP. As shown in Figure 2, even at high concentrations of LCEP ranging from 10 mg/mL to 80 mg/mL, the extracts did not show any inhibitory effects on cell viability (Figure 3A). Similarly, another assessment using human immortal fibroblast cell line, NIH-3T3 cell, exhibited similar results (Figure 3B) that were consistent with those using the HaCaT cells.

### 2.3. In Vivo Evaluation of LCEP On Wound Healing Process

**Figure 4:**
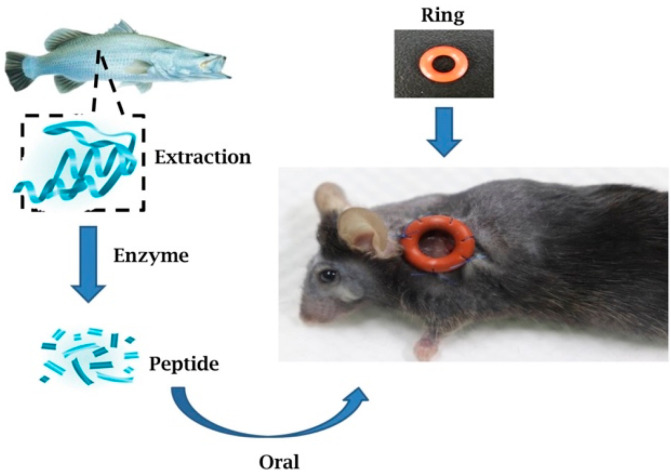
Schematic diagram of the in vivo study.

Mouse wounds are not the same as human wounds in terms of wound healing mechanisms. Mice wounds heal primarily by the contraction of the epidermis, while human wounds heal mainly depending on the regeneration of granulation tissue [18]. In order to properly simulate human wounds in mice, we must overcome the problem of excessive contraction of the wound epidermis [21,22,23,24]. To overcome this problem, after creating a wound on mice, we stitched a silicone ring around the wound to inhibit excessive contraction of the wound epidermis [21]. This splinting model has been proved to decrease the contracting effect and significantly decelerated the wound heal process in rodents [25]. The schematic diagram of the in vivo study using the mouse excisional wound splinting model is shown in Figure 4.

**Figure 5:**
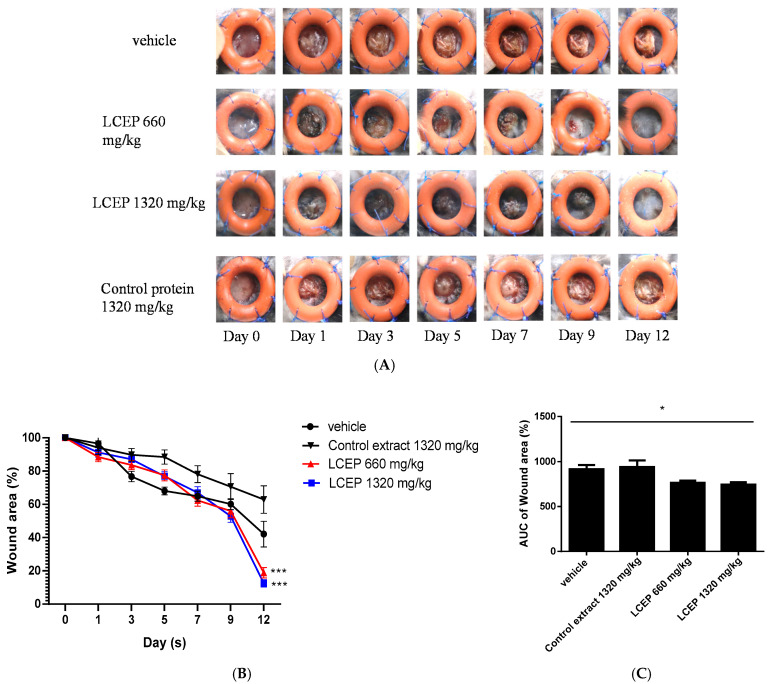
Effects of oral LCEP treatment on wound healing in murine wound model using splint rings. LCEP and control protein were given daily to mice after the surgery for 12 days. Photos of the wounds were taken from day 0 to day 12 (**A**). The percentage of the wound area was calculated (**B**). Area under the curve (AUC) of the wound area were calculated (**C**). Data are means ± standard deviation (SD) (*n* = 9). The differences between the treatment groups and the vehicle group were analyzed using one-way ANOVA followed by the post hoc Dunnett’s multiple comparisons test. Significant thresholds were set at *** *p* < 0.001 (**B**) and * *p* < 0.05 (**C**).

After creating the wound splinting model, mice were orally given LCEP 660 mg/kg and 1320 mg/kg daily for 12 days. We also fed a group of mice with control protein, bovine serum albumin, 1320 mg/kg daily. The photos of wounds were taken on days 1, 3, 5, 7, 9 and 12. Data showed that mice treated with LCEP 660 mg/kg and 1320 mg/kg healed faster than mice treated with vehicle solution or control protein (Figure 5A). Morphological evaluation was conducted by calculating the percentage wound area (WA%). Starting from day 5, the wound areas in LCEP groups were smaller than those in control groups. Further, at day 12, the *p* values of the significant difference between LCEP groups and control group are less than 0.001. (Figure 5B,C). It seems that oral administration of LCEP accelerates wound closure. However, the underlying mechanisms of LCEP induced wound healing remains elusive.

### 2.4. The Effects of LCEP on the Formation of Microvessel

**Figure 6:**
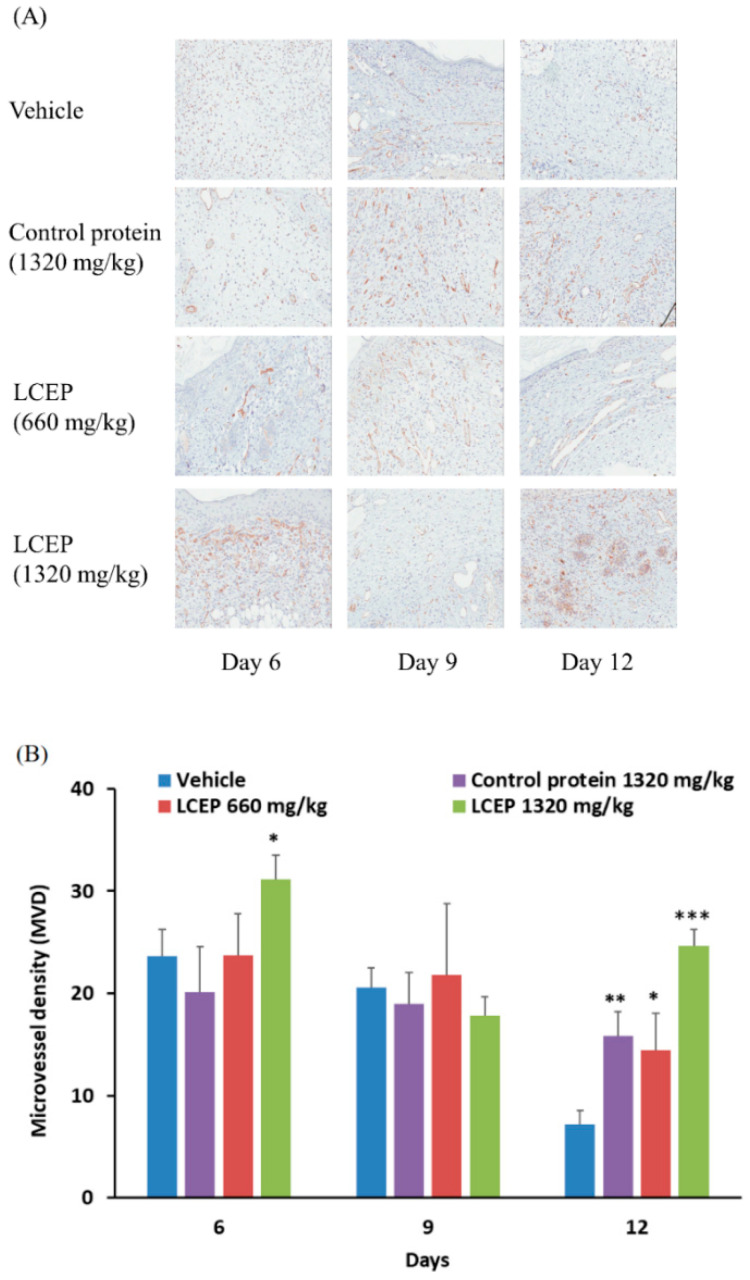
Effects of oral LCEP treatment on angiogenesis in murine wound model using splint ring. Immunohistochemistry analysis was applied to investigate the microvessel formation in mice. Mice fed with LCEP for 6 days, 9 days or 12 days, after which they were sacrificed and subjected to CD31 staining in order to evaluate the status of angiogenesis (**A**). The quantitative data of microvessel density (MVD) were analyzed by CD31 immunohistochemistry staining (brown). Density was analyzed by staining area (mm^2^) using ImageScope 12.4 (**B**). Data are means ± standard deviation (SD) (*n* = 7). The differences between treatment groups and vehicle groups were analyzed using one-way ANOVA followed by the post hoc Dunnett’s multiple comparisons test. Significant thresholds were set at * *p* < 0.05, ** *p* < 0.01, and *** *p* < 0.001.

Based on the morphological evaluation of the wound area, oral administration of LCEP improved wound healing and increased granulation tissue generation. The formation of a new microvessel system is important in tissue regeneration and wound healing. It allows the transport of sufficient nutrients through blood vessels and, thus, for the repair of wounds. Therefore, we further investigate the role of angiogenesis in LCEP-improved wound healing in mice. CD31 expression is a dominant indicator for detecting angiogenetic activity and can be used to detect microvessel in tissue [26]. To investigate the effect of LCEP on the formation of microvessels, CD31 expressions were detected in wound tissue at day 6, day 9 and day 12 (Figure 6A). The quantitative data of microvessel density (MVD) were calculated in CD31 stained tissue. Results showed that LCEP 1320 mg/kg treatment significantly increased the MVD at day 6. On day 12, MVD was significantly higher in control protein, LCEP 660 mg/kg, and LCEP 1320 mg/kg group compared to vehicle group. The high concentration of LCEP shows a more potent effect compared to control protein and low concentration LCEP. Angiogenesis is one of the most important factors for wound healing. Based on our results, it is suggested that oral administration of LCEP effectively enhances wound healing through stimulating angiogenesis at least partially.

## 3. Discussion

The wound in tissues and skins breaks the first line of defense and becomes the hotspot for entering pathogens in the organism. The management of wound remains challenging for clinicians as it cost a lot to the healthcare system. Most of the drugs available in the market, such as cytokines, immunomodulatory factors, and other growth factors, did not meet the ideal clinical requirement [11,27,28]. These drugs have some side effects, which cannot be evaded for some patients. In addition, some antibiotics, such as ampicillin, gentamicin, and rolitetracycline, may reduce wound tensile strength and disrupt collagen organization [29,30]. These prompted scientists to explore natural products, which can meet clinical efficiency with fewer side effects. In recent years, large amounts of natural products have been extracted by using different methods. These products consist of active compounds that have different efficacy, such as anticancer [31,32], anti-inflammation [33,34], and regulation of angiogenesis [10,35,36]. It shows that natural products are the potential source for drug discovery. Bioactive peptides are known for their preventive action against pathogenic species and the treatment of a plethora of diseases. The highly active, stable and target-specific bioactive peptides drive global scientific consideration to get an insight into its wound healing property [37,38]. To date, many bioactive and synthetic peptides are characterized for their wound healing potencies, such as collagen peptides derived from jellyfish, peptides from golden cross band frog and many more [39,40].

Sea bass is used for food in the daily lives of many people, and in some countries, people have bass soup after surgery in the hope that the soup will make the wound heal faster. However, the mechanism by which sea bass aids in wound healing was not clear. A recently published research article has reported the effect of sea bass extract on wound healing, mentioned that sea bass extracts are mainly used to reduce the inflammatory response to help the wound heal [4]. Inspired by their research that sea bass plays a crucial role in wound healing, we have designed our study to establish a model of rodent in wound healing and to compare the differences between enriched small molecule peptides from sea bass extract and from control protein and their efficacy in wound healing. We have used a splint ring to nullify the excessive contraction of the wound epidermis, which aids in mimicking human skin architecture (Figure 4). As we know, apart from sharing a number of similarities in terms of physiology and biochemistry, many contrasting features work well in the mice model but fail to perform in humans. The key differences between mice and human skin architecture are its thickness; human skins are thicker, as the epidermis is composed of 5–10 layers, mice have a relatively 2–3 layer of the epidermis. The unique panniculus carnosus layer present in mice stimulates closure of the wound after injury. Lesser thickness also promotes percutaneous absorption, which led to a faster contraction of the wound in mice [23,24]. Human wounds healing involves mainly re-epithelialization and granulation tissue regeneration. The splint ring we placed in such a way in our murine model after wound creation that it inhibits wound edge contracture and repeats the human mechanism of injury repair [25,41].

In the present work, we have observed that the traditional sea bass soup and sea bass extracts are significantly different in terms of their peptide distributions. The peptides in sea bass soup are mainly distributed in the 1000~12,000 mass (*m*/*z*) range, while those in sea bass extract are mainly in the 1000~5000 mass (*m*/*z*) range (Figure 2). Moreover, another uniqueness in sea bass extract in comparison to sea bass soup is the prevalence of small peptides. We then performed in vitro experiments using HaCaT and NIH3T3 cells to analyze the cytotoxicity of the sea bass extract. It was found that the sea bass extract had no cytotoxic effect at a low dose of 10 mg/mL or a high dose of 80 mg/mL (Figure 3). This encourages us it is safe to use in the animal system to investigate its wound healing efficiency in in vivo models. After obtaining this cytotoxicity data, we directly carried out in vivo experiments on mice. We found our enzymatically derived sea bass extract very promising in contracting the wound area, as reported for previous bioactive peptides from amphibian species [11].

Because the content of sea bass soup is edible, we conducted a tube feeding experiment, sea bass extract fed to mice in order to distinguish whether its nutrients accelerate wound healing while using control protein as a positive control for nutrients (Figure 5 and Figure 6). The results showed that both the LCEP and control protein could aid wound healing, the regeneration of granulation tissue, and angiogenesis. The effect of control protein was mainly concentrated in the early stages of wound healing, and the wound healing rate was decreased in the later stage. The LCEP had noticeable effects on wound healing at doses of 660 mg/kg and 1320 mg/kg body weight. The morphology analysis clearly showed the wound closure becomes faster with LCEP treatments (Figure 5). The contraction of the wound area indicates re-epithelization and formation of granulation tissues.

CD31 is a well-established marker for angiogenesis [42]. CD31 expression was screened during the process of LCEP treatment, as can be seen in Figure 6. Mice fed with a high dose of LCEP showed increased CD31 signal [43]. This explains that LCEP accelerates the healing of injury by increasing vascularization around the injury site. The findings of the study are comparable to the previously studied work by Song Yongli et al., 2018 evaluation of collagen bioactive peptides from jellyfish in wound healing in mice models [11,43]. The wound healing property of collagen peptides isolated from Nile tilapia skin carried out in rabbits was found similar. The exact mechanism through which peptide extract accompanied the healing process is still needed investigation. In the future, we will conduct more research in this area in the hopes of making additional contributions to the study of wounds and their therapeutic development.

## 4. Materials and Methods

### 4.1. Lates Calcarifer Sample Preparation

The raw material consisted of sea bass skin, scales, bones, and meat. The raw material was washed 2 to 5 times with reverse osmosis (RO) water to remove blood and impurities, thereby obtaining a prepared bass sample. The control extracts of sea bass were prepared by the hot boiling water. The sea basses were extracted by hot boiling water for 1 to 5 h, and then the extract was lyophilized.

Next, the prepared bass sample was treated with an acid solution of 1:5 (*w*/*v*), in which the concentration of the acid solution was 1% to 5% hydrochloric acid. The acid-treated sample was immersed in water with a temperature of 15 °C to 20 °C for 10 to 48 h; then, the acid-treated sample was washed 2 to 5 times with RO water to remove the acid solution before finally being adjusted with calcium carbonate. The pH of the ultimately obtained mixture was between 4 and 8.

The mixture was extracted with hot water with a temperature of 55 °C to 100 °C for 1 to 6 h before being centrifuged at 3000 rpm for 10 min. Next, the impurities were filtered. Purification with 0.2% to 1% calcium carbonate or limestone was followed by filtration through a 1 to 10 μm filter and an ion exchange resin column to remove impurities and the limestone, further separating and purifying the effective peptide extraction mixture. The active peptide was then treated with activated carbon to deodorize and decolorize it, and then its volume was concentrated to 1/10 to 1/20 at 50 °C to 60 °C.

Next, the effective peptide was digested with an enzyme complex at 40 °C to 60 °C for 1 to 5 h to obtain a digested product. After digestion, the enzyme in the digestion product was denatured at 85 °C to 95 °C for 10 to 30 min and then cooled. The mixture was filtered twice with diatomaceous earth and activated carbon; the digested product was sterilized at an ultra-high-temperature for 3 to 5 s, and the peptide of the sea bass extract was obtained by filtration through a 0.2 μm filter.

### 4.2. Lates Calcarifer Molecular Weight Assay

The sample was centrifuged at 13,000 rpm. After 2 min, we collected 200 μL supernatant, and the supernatant was aspirated. The supernatants were desalted and concentrated by using a C18-ZipTip (Millipore). The samples were dissolved in water and assayed using an Applied Biosystems Voyager-DE PRO (MALDI-TOF).

### 4.3. Cell Culture

Referring to the improved method reported by Jawun Choi et al. [20]. Human keratinocyte HaCat and mouse embryonic fibroblast NIH3T3 cells were cultured in Dulbecco’s modified Eagle’s medium (DMEM) containing 10% fetal bovine serum (FBS), penicillin (5%), and streptomycin (5%) at 37 °C in an incubator containing 5% CO_2_.

### 4.4. Cell Viability Assay

Referring to the improved method reported by Kuen-Ming Wu et al. [44]. The viability of the HaCat cells and NIH3T3 cells following treatment with the *Lates calcarifer* extract was determined by MTT assay. The cells were grown in 96-well plates and treated with 10, 20, 40 and 80 mg/mL of *Lates calcarifer* extract for 24 h. After adding 100 μL of DMEM and 20 μL of a 2.5 mg/mL MTT solution, the cells were incubated in a humidified atmosphere of 5% CO_2_ at 37 °C for 1 h living cells, and MTT will combine into purple crystals, and then the purple crystals were dissolved in 50 μL of dimethyl sulfoxide (DMSO). A photometer was then used to measure the absorbance at a wavelength of 575 nm.

### 4.5. Animals

Thirty-four 5-week-old wild-type C57BL/6 J mice (BioLASCO Taiwan Co., Ltd., Taiwan) were used in the animal experiments. The mice were kept one mouse per cage and before and after surgery in a temperature-controlled facility under a 12 h light/dark cycle. The mice were allowed to eat food and water. All mice were separated into four groups: vehicle (*n* = 9), control extract (*n* = 9), LCEP low dose (*n* = 9), and LCEP high dose (*n* = 9). Mice were fed with vehicle (water), control extract (1320 mg/kg), LCEP low dose (660 mg/kg), and LCEP high dose (1320 mg/kg) for 12 days. All animals were housed in a clean conventional area, and the experimental protocols were performed and approved by the Institutional Animal Care and Use Committee (IACUC) of China Medical University guidelines. Each group (*n* = 3) was sacrificed after 6 days, 9 days, and 12 days using carbon dioxide asphyxiation according to institutional guidelines.

### 4.6. Splints

Referring to the improved method reported by Marshall CD et al. [25,41]. Splints were made of round silicone (with each piece of silicone having a 6 mm internal diameter). All of the procedures and tools were performed/used under sterile conditions. Anesthetized mice were treated with isoflurane. For each mouse, after removing the back hair with an electric razor, a round wound (6 mm) was made on the back, with the skin that had covered the wound area being removed. The silicone ring was then placed around the wound and fixed to the skin with a surgical line (6-needle suture).

### 4.7. Morphological Analysis

All of the photos of each wound, including the unpeeled area, were loaded into Image J. The tissue was identified with the naked eye. We compared the healing areas in the silicone ring. Specifically, the wounds of mice treated with the sea bass extract and a control group were compared to determine whether they exhibited any statistically significant differences. Image J calculations were performed to compare the healing rate of each wound on each day, the average scar size, and the scar size ratio for the groups. The following equation was applied to evaluate the wound healing rate. WA% = wound area at the time of measurement/initial wound area × 100

The length of data for healing the wound was then searched for the area under the curve (AUC), namely the area under the curve between the average length of wound healing for the time of observation. AUC is calculated from the average wound length of day 0 to day 12. Calculation of AUC values with the trapezoid method:

$${\left[\mathrm{AUC}\right]}_{t_{1}}^{t_{7}}=\overset{7}{\underset{n=1}{\sum }}\left\{\frac{P(t_{n})+P(t_{n-1})}{2}\times \left(t_{n}-t_{n-1}\right)\right\}$$

Let *tn* be defined as which day the data was taken: *n* = 1, Day = 0; *n* = 2, Day = 1; *n* = 3, Day = 3; *n* = 4, Day = 5; *n* = 5, Day = 7; *n* = 6, Day = 9; *n* = 7, Day = 12. Let *P(_tn_)* be defined as the 100% minus the healing percentage of the wound of the day.

### 4.8. Immunohistochemical Staining

After surgery, the mice were euthanized on the 6th, or 9th and 12th day, respectively and the silicone ring was removed along with the skin. The skin tissue was then fixed in a 30% formalin solution overnight and then processed. We used a pressure cooker for antigen retrieval. The anti-CD31 primary antibody for recognizing CD31 (Abcam; P2B1) was applied to IHC with a dilution of 1:500. The reaction of tissue and antibody were performed by antibody Amplifier Quanto for 12 min, and then it was reacted with HRP Polymer Quanto for 10 min. The DAB Quanto Chromogen/substrate was reacted with slides and washed with distilled water. For the analysis of microvessel density, the expression of CD31 was conducted to evaluate the microvessel density by using immunohistochemistry of skin tissue. ImageScope 12.4 (Aperio; Leica Biosystems) analysis was used to assess the microvessel density. The microvessel density was imaged and counted by analyzing the total area, and each microvessel density was selected from three areas of wound area according to the method described [3].

### 4.9. Statistical Analysis

All the experiments are performed independently three times. Data in results represented are standard deviations. Statistical significance of the variables was done by Student’s *t*-test. A *p*-value of *p* < 0.05 was considered statistically significant.

## 5. Conclusions

In summary, control protein and sea bass peptide extract LCEP can indeed help wounds heal to a significant extent, and the effect of LCEP in this regard is better than that of sea bass soup. This may be because of the abundance of small peptides in LCEP, which makes it easier to absorb and thus accelerate wound healing. The in vivo experiment result on mice wound area measurement and immunohistochemistry results confirmed the wound healing potency of LCEP. However, our study did not demonstrate the direct mechanism of wound healing by this peptide extract. To determine the exact pathway requires more research. Chronic wounds led to excessive inflammatory reactions, which cause adverse effects against angiogenesis. From previous studies, we learned that *Lates calcarifer* soup could reduce inflammation and aid wound healing. In this study, we found that LCEP can improve angiogenesis. Our investigation strongly suggests that LCEP may be helpful in the treatment of chronic and acute wounds if included in appropriate nutritional supplements.

## Data Availability

Not applicable.
